# A Landscaping Assessment and Call‐to‐Action to Improve Access to Novel Reserve Antibiotics in 14 Low‐ and Middle‐Income Countries

**DOI:** 10.1002/puh2.70005

**Published:** 2024-10-10

**Authors:** Fabrizio Motta, Summiya Nizamuddin, Ejaz Khan, Tracie Muraya, Silvio Vega, Joseph Fadare, Shaffi F. Koya, Maria Virginia Villegas, Faisal Sultan, Tara Lumley, Rahul Dwivedi, Lauren Jankelowitz, Jennifer Cohn

**Affiliations:** ^1^ Santa Casa de Misericórdia de Porto Alegre Porto Alegre Brazil; ^2^ Shaukat Khanum Memorial Cancer Hospital and Research Centres (SKMCH&RC) Lahore Pakistan; ^3^ Shifa International Hospital Shifa Tameer‐e‐Millat University Islamabad Pakistan; ^4^ REACT Africa Lusaka Zambia; ^5^ Complejo Hospitalario Metropolitano Caja de Seguro Social Sistema Nacional de Investigadores (SNI) Panama City Panama; ^6^ Department of Pharmacology and Therapeutics Ekiti State University/Ekiti State University Teaching Hospital Ado‐Ekiti Nigeria; ^7^ Global Institute of Public Health Thiruvananthapuram Kerala India; ^8^ Grupo de Investigaciones en Resistencia Antimicrobiana y Epidemiología Hospitalaria (RAEH) Universidad el Bosque Bogotá Colombia; ^9^ L.E.K. Consulting Group London UK; ^10^ Global Antibiotic R&D Partnership (GARDP) Geneva Switzerland

**Keywords:** access, antimicrobial resistance, low‐ and middle‐income country (LMIC), Reserve antibiotics

## Abstract

**Background:**

Antimicrobial resistance (AMR) presents a significant global mortality burden which particularly affects the low‐ and middle‐income countries (LMICs). Enhancing diagnostics to identify drug‐resistant infections and improving appropriate access to novel Reserve antibiotics in LMICs can address AMR‐related morbidity, mortality and healthcare costs. This article characterizes the AMR landscape across 14 LMICs and describes an introductory pathway for novel Reserve antibiotics.

**Methods:**

This mixed‐method study was completed in 14 LMICs in Africa, the Americas, Asia and Europe through a combination of qualitative interviews with physicians and public health experts (PHEs), and a quantitative survey of physicians, supported by an assessment of secondary materials relating to antibiotic introduction and AMR burden.

**Results:**

A total of 54 physicians and 17 PHEs were interviewed, and 209 physicians participated in the survey. Top unmet needs across public and private settings were as follows: access to new antibiotics to better manage drug‐resistant infections; affordability; adequate safety profile for prescribed antibiotics. Access to diagnostics and antibiotic susceptibility testing was noted as a barrier, with large tertiary and private centres experiencing better access. Implementation of antibiotic stewardship programmes was variable and limited by insufficient funding, shortage of infectious disease physicians, poor AMR education and lack of restrictions to limit antibiotic use. Antibiotic access varies by sector, centre type, location and strength of individual state procurement systems. In particular, private sector facilities have better access to Reserve products. In most countries, most Reserve antibiotics included in WHO's Essential Medicines List (EML) were not included in national EMLs or not registered in countries.

**Conclusion:**

This study has helped to identify common barriers and pathways to Reserve antibiotic access, irrespective of the level of preparedness of countries. The data offer insights into possible solutions to improve access and highlight opportunities to strengthen access pathways and expedite access, for example, by identifying priority antibiotics based on national public health need. A six‐step introductory pathway for novel Reserve antibiotics is described.

## Graphical abstract

Antimicrobial resistance (AMR) is a major global health issue, especially in low‐ and middle‐income countries (LMICs). This study explores the AMR landscape in 14 LMICs, highlighting barriers to accessing novel Reserve antibiotics, such as affordability, safety and diagnostics. Despite varying access to antibiotics across different sectors, the study identifies pathways to improve access, emphasizing the need for national prioritization of essential antibiotics and suggesting a six‐step introduction process for novel Reserve antibiotics.

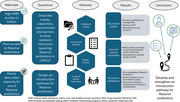

## 1 Introduction

Antimicrobial resistance (AMR) presents a significant global mortality burden, directly leading to 1.27 million deaths in 2019, making it the third most common underlying cause of death worldwide, following only stroke and ischaemic heart disease [1, 2]. The highest mortality rates are found in low‐ and middle‐income countries (LMICs) [1, 3]. Across the world, infection rates are expected to continue to rise, and by 2050, 4.1 and 4.7 million annual infection‐related deaths are predicted in Africa and Asia, respectively [4].

Several global initiatives have been launched to combat the growing burden of AMR. In 2017, the WHO launched the AWaRe Classification of Antibiotics. The AWaRe classification categorizes antibiotics into three groups – Access, Watch and Reserve – and aims to optimize the quantity and quality of antibiotic prescribing [5]. Reserve antibiotics are last‐resort antibiotics that should only be used to treat severe infections caused by multidrug‐resistant pathogens [5, 6].

In geographic areas and populations where antibiotic resistance is growing, it is important to improve appropriate access to diagnostics and Reserve antibiotics to address the morbidity, mortality and healthcare costs associated with AMR. Although LMICs face disproportionate burden of resistant bacterial pathogens, including those on the WHO Priority Pathogen List [6], these same countries often have limited access to novel antibiotics that target resistant infections [7]. Although a number of studies have summarized current work on AMR National Action Plans or stewardship programmes, there is not information in the literature about the process to introduce novel antibiotics in LMICs [8, 9]. The lack of literature on this topic is in contrast to other disease areas, such as HIV or tuberculosis, where significant literature on introduction of novel medications exists [10, 11]. For novel Reserve antibiotics to be introduced appropriately in LMICs, the AMR landscape into which they are being introduced must be better understood, and the introductory pathway built with the leadership of local experts and the government. This article aims to characterize the local AMR landscape across 14 LMICs, including access to diagnostics and antibiotics, focussing on Reserve antibiotics and map potential introductory pathways for novel Reserve antibiotics.

## 2 Methods

### 2.1 Study Design and Setting

This is mixed‐method study, completed in 14 LMICs in Africa, the Americas, Asia and Europe. Countries were selected on the basis of [1] whether the country has identified AMR as a priority and has some governance structure and/or a plan to address AMR (as evidenced by having an AMR National Action Plan developed or implemented according to the WHO Global Database for Tracking AMR Country Self‐Assessment Survey [12]) [2], whether the country has documented evidence of significant AMR by prevalence of carbapenem‐resistant (CR) organisms (>20% CR organisms in surveillance samples, such as WHO surveillance [13]) or is very likely to have (as evidenced by surrounding countries or evidence from other non‐GLASS literature [14]) [3], selection of countries to provide geographic diversity and representation. A greater number of interviews were conducted and survey responses collected for seven countries (South Africa, India, Mexico, Brazil, Jordan, Egypt and Kenya), and fewer interviews conducted and survey responses collected for seven other countries (Georgia, Pakistan, Guatemala, Nigeria, Colombia, Panama and Serbia) (Table 1). The same topics were covered for all countries.

**TABLE 1:** Study participants and characteristics – qualitative research.

			Qualitative research	
			Physicians	Public health experts
Total			54	17
**By country and profession**	Detailed assessment	South Africa	14	2
		India	5	1
		Mexico	5	2
		Brazil	4	—
		Jordan	2	1
		Egypt	4	—
		Kenya	2	4
	Higher level assessment	Georgia	2	2
		Pakistan	3	1
		Guatemala	3	—
		Nigeria	4	2
		Colombia	3	1
		Panama	1	1
		Serbia	2	—
**By sector (%)**	Public		76%	n/a
	Private		24%	n/a
**By professional experience (%)**	Infectious disease specialist		70%	n/a
	Non‐specialist consultant (general hospital‐based physicians, surgeons, neonatologists, paediatricians etc.)		15%	n/a
	Medical microbiologist		7%	n/a
	Critical care specialist		8%	n/a
	Internal medicine		n/a	n/a
**By primary treatment setting (%)**	Primary and secondary centres		n/a	n/a
	Tertiary centres		n/a	n/a

#### 2.1.1 Data Collection

##### 2.1.1.1 In‐Depth Interviews

The research employed in‐depth qualitative semi‐structured interviews of 60‐min duration. Participants considered were physicians and public health experts (PHEs). The consulting firm L.E.K. was contracted to conduct, transcribe and summarize the expert interviews.

The selection of study participants was a mix of targeted and opportunistic and focused on selecting individuals on the basis of evidence of participation in initiatives related to AMR (e.g. sponsors or stewards of NAPs or national antimicrobial stewardship programmes [ASPs]) or participation in conferences or events related to AMR, authorship of AMR‐related peer‐reviewed publications or guidelines. Once solicited, experts had to fulfil the following criteria: (1) work at a tertiary, private or university hospital, or a research centre or centre for public health; (2) score 3 or more (out of 5) in comfortability discussing antibiotic introduction and AMR burden; (3) be active in initiatives related to AMR (e.g. involvement in clinical trials, conferences, guidelines development or stewardship programmes). Finally, experts who were practicing physicians needed to treat 50 or more patients with AMR bacterial infections per month.

##### 2.1.1.2 Survey

This research also employed an anonymous cross‐sectional survey of 209 physicians. The online questionnaire was informed by findings from the qualitative interviews and assessment of secondary materials. The questionnaire was developed in English and translated into Arabic, Portuguese, Spanish, Georgian and Serbian, then edited for accuracy and local appropriateness. It was piloted in each of the 14 LMICs and took 20 min to complete. Experts were screened and selected according to the same criteria as those involved in the qualitative research. The recruitment spanned 3 months, from June to August 2022. The consulting firm L.E.K. was contracted to develop and distribute the questionnaire and analyse the results.

The survey collected information on participant demographics, access to microbiologic and antibiotic sensitivity testing, availability or treatment guidelines, presence and implementation of ASPs, drivers of antibiotic selection and availability and barriers to access to prescribed antibiotics. The survey also asked respondents to rate key unmet needs for patients with carbapenem resistant infections on a Likert scale, with 1 corresponding to ‘low’, 2 to ‘medium’ and 3 to ‘high’.

Survey participants were also asked which diagnostic testing methods were currently available in their centres. This study focused on availability and did not examine practices of use of diagnostic/sensitivity tests or barriers to use (such as patient out‐of‐pocket payment) for such tests. The study also did not specify whether the diagnostics were available on site or by sample or patient referral.

##### 2.1.1.3 Document Analysis

In parallel, secondary materials relating to antibiotic introduction and AMR burden were assessed. Key international websites and resources, such as GLASS, WHO and Organization for Economic Co‐operation and Development (OECD), were reviewed for information on AMR. Additionally, national sources, such as the ‘AMR Research and Surveillance Network’ annual report, published by the Indian Council of Medical Research in 2021 were also reviewed [15]. This was coupled with other national website for the latest available information on antibiotic treatment guidelines, AMR National Action Plans and national ASPs.

##### 2.1.1.4 Review

A semi‐structured literature search on PubMed from 2010–2022 was completed to collate papers related to AMR epidemiology, antibiotic usage and procurement in target countries. Key search terms included: ‘antimicrobial resistance’; ‘AMR’; ‘local resistance patterns’; ‘LMICs’; ‘low‐ and middle‐income countries’; ‘antibiotics’; ‘Reserve’; ‘Access’.

##### 2.1.1.5 Data Analysis

A thematic analysis of the interview data in English was conducted using the framework method [16]. Two analysts independently reviewed all transcripts, then met to discuss any differences in their reviews, come to consensus and identify key themes that emerged from the data. Key themes were reviewed and agreed upon by the interviewer.

The survey data were cleaned to remove any errors, inconsistencies or incomplete responses and then analysed descriptively in Excel and Qualtrics. Data from each of the research methods were analysed separately and then integrated, using the qualitative findings and assessment of secondary materials to explain the quantitative survey results.

##### 2.1.1.6 Ethical Considerations

This investigation did not meet the criteria for human subjects research as information collected from key informants was limited to enquiries around health system functioning and not personal information. However, informed consent was obtained from all persons interviewed and surveyed.

## 3 Results

### 3.1 Profile of Participants

#### 3.1.1 Qualitative Study Participants

One hundred and sixty‐six experts were invited to participate in the qualitative research, and 71 experts accepted. Of the 71 experts interviewed, 76% were healthcare professionals (HCP) and 24% PHEs, defined as non‐clinical experts with experience in AMR. Of the HCPs, 63% worked in public facilities and the remainder in the private sector (Table 1). In these, 70% of the HCPs were ID specialists, and others included critical care specialists (8%), general hospital‐based physicians – for example surgeons, neonatologists and paediatricians – (15%) and medical microbiologists (7%). Among these, 93% of the HCPs focused on adult patients, whereas 7% had a paediatric focus (Table 1).

#### 3.1.2 Quantitative Study Participants

Of the 209 experts surveyed, 53% worked in primary and secondary centres and 47% in tertiary centres; 51% in private centres and 49% in public; 49% of experts were non‐specialist consultants or general hospital‐based physicians – for example surgeons, neonatologists and paediatricians; 25% ID specialists, 11% critical care specialists and 13% internal medicine (Table 2).

**TABLE 2:** Study participants and characteristics – quantitative survey research.

			Quantitative research
			Physicians
Total			209
**By country and profession**	Detailed assessment	South Africa	20
		India	20
		Mexico	20
		Brazil	20
		Jordan	20
		Egypt	20
		Kenya	20
	Higher level assessment	Georgia	10
		Pakistan	10
		Guatemala	9
		Nigeria	10
		Colombia	10
		Panama	10
		Serbia	10
**By sector (%)**	Public		49%
	Private		51%
**By professional experience (%)**	Infectious disease specialist		25%
	Non‐specialist consultant (general hospital‐based physicians, surgeons, neonatologists, paediatricians etc.)		49%
	Medical microbiologist		1%
	Critical care specialist		11%
	Internal medicine		13%
**By primary treatment setting (%)**	Primary and secondary centres		53%
	Tertiary centres		47%

## 4 AMR Management Landscape Assessment

The AMR landscape was reviewed for each country, including the level of unmet need, access to microbiologic and antibiotic sensitivity testing, presence and implementation of ASPs, availability and barriers to access to prescribed antibiotics.

### 4.1 Unmet Needs

The top three unmet needs for patients with CR infections were the same across public and private settings and included access to new antibiotics to better manage drug‐resistant infections (public, average 2.4 [*n* = 99], private 2.1 [*n* = 101]), better affordability (public 2.3 [*n* = 99], private 2.1 [*n* = 101]) and improved safety/toxicity profile (public 2.1 [*n* = 99], private 2.0 [*n* = 101]) of these antibiotics (Table 3). Public sector experts reported higher overall levels of unmet need versus private sector experts across all countries included in this study (Table 3). Physicians in Pakistan reported the highest levels of unmet need (Table 3). Other high‐ranking unmet needs included quality assurance of treatment options, more rapid and accurate diagnostics, better testing for CR specifically, better infection control and improved efficacy and response to currently available treatment options.

**TABLE 3:** Unmet needs for patients with carbapenem‐resistant infections (1 – low unmet need; 3 – high unmet need).

	By sector		By country													
Unmet need	Private	Public	South Africa	India	Mexico	Brazil	Jordan	Egypt	Kenya	Georgia	Pakistan	Serbia	Guatemala	Nigeria	Colombia	Panama
Stable supply of essential antibiotics	1.6	1.7	1.8	1.6	1.2	2.0	1.7	1.4	1.4	2.6	2.5	1.6	2.3	1.8	1.4	1.7
Infection control	1.8	1.9	1.9	1.7	1.8	2.1	2.0	1.9	2.0	2.0	2.2	1.9	1.8	1.9	1.6	1.8
New products that better manage carbapenem‐resistant infections	2.1	2.4	2.4	2.0	2.1	2.1	2.2	2.5	2.4	2.5	2.7	2.1	2.6	3.0	2.3	2.7
Improved efficacy and response to currently available treatments	1.8	1.9	2.0	1.6	2.0	1.9	2.0	1.9	1.9	2.2	1.9	1.8	2.0	1.9	1.8	1.9
Convenient frequency of administration (i.e. improved dosing schedule due to longer in vivo half‐lives)	1.7	1.8	1.8	1.8	1.5	1.9	1.7	1.4	1.6	1.9	2.9	1.7	1.8	1.6	1.4	2.0
Better price/affordability	2.1	2.3	2.0	1.6	2.3	2.3	2.5	2.3	2.5	1.9	2.0	2.4	2.8	2.4	2.3	2.4
Improved safety and toxicity (e.g. fewer incidences of diarrhoea, constipation, rash, cough, headache and nausea)	2.0	2.1	2.2	2.1	1.9	2.0	2.3	2.1	2.3	1.9	2.3	1.9	2.2	2.1	1.9	2.1
Accurate early diagnosis of bacterial infection (including cost and availability of tests; speed of receiving results; expertise to accurately interpret test results)	1.7	1.8	1.8	1.7	1.8	1.9	1.8	1.6	1.8	1.9	2.6	1.5	2.3	1.7	1.5	1.8
Testing for carbapenem resistance	1.7	2.0	2.1	1.7	1.7	2.0	1.8	1.6	1.9	1.9	2.7	1.9	2.1	1.7	1.8	1.8
Quality assurance of treatment options	1.8	2.0	2.0	1.9	2.0	1.9	1.8	1.9	2.0	1.9	2.7	1.9	1.3	1.7	1.7	2.0

### 4.2 Diagnostic and Susceptibility Testing

The most widely available standard diagnostic tests reported by experts included Gram staining: average: 98% in private (range: 88%–100%, [*n* = 95]), 96% in public (67%–100%, [*n* = 96]); and polymerase chain reaction (PCR): 94% in private (78%–100%, [*n* = 95]), 84% in public (56%–100%, [*n* = 96]). Access and use of more complex diagnostic tests were more variable, with experts from 9 of 14 (64%) countries reporting the availability of nanoparticle probe technology, and peptide nucleic acid fluorescence in situ hybridization (PNA‐FISH), and 6 countries reporting the availability of isothermal nucleic acid amplification technology (INAAT). The least widely available diagnostic tests reported by experts included PNA‐FISH: 15% in private (0%–50%, [*n* = 95]), 18% in public (0%–50%, [*n* = 96]); and INAAT: 8% in private (0%–38%, [*n* = 95]), 15% in public (0%–56%, [*n* = 96]).

Clinical laboratories currently use several identification and antimicrobial sensitivity testing (AST) methods. Conventional AST methods include culture‐dependent methods (e.g. a disk diffusion test and broth microdilution), molecular‐based methods (e.g. PCR) and more advanced technologies such as whole genome sequencing, matrix‐assisted laser desorption ionization‐time of flight (MALDI‐TOF) and INAAT [17]. Access and uptake of AST are variable across the countries assessed. Thirteen of the 14 countries reported access to standard AST methods such as broth microdilution, disk diffusion and PCR. The most widely available AST methods reported by experts included broth microdilution: 84% in private (0%–100%, [*n* = 95]), 73% in public (33%–100%, [*n* = 96]); disk diffusion: 85% in private (0%–100%, [*n* = 95]), 77% in public (44%–100%, [*n* = 96]); and PCR: 82% in private (0%–100%, [*n* = 95]), 77% in public (40%–100%, [*n* = 96]). Access and use of more complex AST methods were more variable, with 43% (6 of 14) of countries reporting access to complex tests such as INAAT. The least commonly available AST methods reported included mass spectrometry (e.g. MALDI‐TOF): 36% in private (0%–56%, [*n* = 95]), 30% in public (0%–60%, [*n* = 96]); whole genome sequencing: 26% in private (0%–100%, [*n* = 95]), 16% in public (0%–75%, [*n* = 96]); and INAAT: 11% in private (0%–38%, [*n* = 95]), 18% (0%–44%, [*n* = 96]).

Experts in South Africa, Jordan, Egypt and India reported the most advanced testing landscapes (i.e. reported the most availability of diagnostic and sensitivity tests). Overall, large tertiary centres (where 47% of sample respondents worked) and most private facilities had better access to diagnostic and sensitivity testing, including better access to more complex tests or automated equipment (e.g. MALDI‐TOF). Experts also noted that aside from access to the tools themselves, availability of some materials to support diagnostic and AST is a major issue in LMICs – for example low availability of discs used for susceptibility testing for sulfamethoxazole in Pakistan. Further to laboratory capacity and capability constraints, many laboratories in LMICs, especially in the public sector, lack quality assurance and accreditation.

### 4.3 Antibiotic Stewardship Programmes

Four countries had national ASPs available (South Africa, India, Egypt and Pakistan), eight had ASPs partially available, that is national ASPs available in principle but not implemented (Brazil, Jordan, Kenya, Serbia, Georgia, Nigeria, Colombia and Panama), and two had no presence of national ASPs (Mexico and Guatemala). Limitations to the successful implementation of ASPs were driven by limited funding, a lack of ID physicians, lack of AMR education and lack of restrictions to limit antibiotic use. In countries where ASPs were not available or only available partially, some major tertiary centres did have simplified (e.g. limited to formulary restrictions) ‘in‐house’ ASPs developed locally.

Survey respondents assessed the level of success of their ASPs, which were largely similar across private and public settings. Factors linked to more successful ASPs include better AMR awareness among the practicing physicians, better and closer monitoring of AMR (e.g. presence of electronic health records, which allow accurate tracking of antibiotics usage) and implementation of key initiatives, such as formulary restrictions and pre‐authorization requirements for antimicrobials, ID specialist and hospital stewardship rounds, dose optimization and nursing practices audits.

### 4.4 Availability of and Barriers to Access for Key Antibiotics

Once approved, antibiotic access is impacted by two main drivers. First, ASP restrictions can limit access to antibiotics deliberately to promote appropriate use (e.g. vancomycin through ASP audits or colistin through pre‐authorization requirements). Second, antibiotic access can be limited by other factors such as restrictions unrelated to appropriate use (e.g. limitation of antibiotics included on the Essential Medicines List [EML], cost sensitivity, stock outs and supply chain issues). These antibiotic access limitations and restrictions vary according to the sector (e.g. public vs. private), type of centre (e.g. tertiary vs. quaternary) and location and strength of individual state procurement systems (e.g. urban vs. rural). An analysis of Reserve antibiotics included in the WHO EML shows that very few of these Reserve antibiotics are included in national EMLs or are registered in countries (Table 4).

**TABLE 4:** Reserve antibiotics on countries’ Essential Medicines List (EML) versus those approved by national regulatory authorities.

	EML					Imported/registered				
Country	Colistin Injection	Polymyxin B	Aztreonam	Ceftazidime–avibactam	Fosfomycin injection	Colistin injection	Polymyxin B	Aztreonam	Ceftazidime–avibactam	Fosfomycin injection
Brazil	N	Y	N	N	N	Y	Y	Y	Y	N
Colombia	N	N	N	N	N	Y	Y	Y	Y	Y
Egypt	N	N	N	Y	N	Y	N	Y	Y	N
Georgia										
Guatemala	N	N	N	N	N	N	N	N	N	N
India	N	N	N	N	N	Y	Y	Y	Y	Y
Jordan	Y	N	N	N	N	Y	N	N	N	N
Kenya	Y	Y	N	N	N	N	N	N	N	N
Mexico	N	N	N	Y	N	Y	N	N	Y	Y
Nigeria	N	N	N	N	N	Y	N	N	Y	Y
Pakistan	Y	N	N	Y	Y	Y	N	N	Y	Y
Panama	Y	Y	N	N	Y					
Serbia										
South Africa	N	N	N	N	N	N	N	N	Y	N

*Note:* Green – included in lists or registered; amber – not included in lists or not registered; grey – data not available.

Antibiotics that are commonly included in guidelines to treat frequent infections (e.g. pneumonia, blood‐stream infection, intra‐abdominal infection and urinary tract infection) encountered in health facilities (primarily inpatient) were identified by respondents (Table 4), and availability assessed. Across the 14 LMICs, access challenges were most frequent amongst antibiotics in the Reserve category (Table 5). However, access issues were also identified for antibiotics under Access and Watch categories (e.g. Access – penicillin G, cloxacillin and Watch – carbapenems like meropenem and piperacillin–tazobactam) (Table 5).

**TABLE 5:** Antibiotic availability reported by physicians in the public sector.

Availability of antibiotics that are commonly included in guidelines to treat frequent infections in public facilities								
*Aminoglycoside*	Amikacin	Gentamycin	Plazomicin	Tobramycin				
*Beta‐lactam/beta‐lactamase inhibitors*	Amoxicillin–clavulanate	Ampicillin–sulbactam	Ceftazidime–avibactam	Ceftolozane–tazobactam	Piperacillin–tazobactam	Cefixime–clavulanic acid	Cefoperazone–sulbactam	Amoxicillin–clavulanate
*Carbapenem*	Doripenem	Ertapenem	Imipenem–cilastatin	Meropenem				
*Cephalosporin*	Cefazolin	Cefepime	Cefiderocol	Ceftriaxone	Cefotaxime	Cephalexin	Cefuroxime	Cephalexin
*Fluoroquinolone*	Ciprofloxacin	Moxifloxacin	Levofloxacin	Ofloxacin	Norfloxacin	Gemifloxacin	Nalidixic acid	
*Glycopeptide*	Teicoplanin	Vancomycin						
*Macrolide*	Azithromycin	Erythromycin	Clarithromycin					
*Penicillin*	Amoxicillin	Cloxacillin	Flucloxacillin	Penicillin G	Ampicillin	Dicloxacillin		
*Phosphonic*	Fosfomycin (IV)	Fosfomycin (oral)						
*Polymyxins*	Colistin	Polymyxin B						
*Tetracycline*	Doxycycline	Tigecycline	Minocycline	Eravacycline				
*Other*	Nitrofurantoin	Linezolid	Daptomycin	Metronidazole	Clindamycin	Rifamycin	Fluconazole	Aztreonam
	Trimethoprim‐sulfamethoxazole	Meropenem–polymyxin‐B						

*Note:* Green – good access and no supply issues noted; amber – no or limited access.

Experts from 12 of the 14 countries included in this study (South Africa, Kenya, Jordan, Brazil, Mexico, Guatemala, Colombia, Pakistan, Nigeria, Panama, Georgia and Serbia) highlighted antibiotic access varied according to the sector of the hospital (e.g. public or private). Across countries, private sector facilities had better availability of antibiotics and fewer access restrictions. This is in part because they procure directly from manufacturers or distributors, whereas in the public sector, antibiotics are typically procured through periodic tenders. Antibiotic access can be further limited in public hospitals by which drugs are included in the EML. Restricted access or lack of EML inclusion due to high price is a barrier, particularly in the case of drugs such as ceftazidime–avibactam, daptomycin and tigecycline. However, access to last‐line antibiotics (e.g. colistin) was also often highly controlled in public hospitals as part of ASPs.

Experts from 7 of 14 countries (India, Egypt, Kenya, Serbia, Pakistan, Georgia and Panama) highlighted antibiotic access also varied according to the level of care (e.g. secondary or tertiary) and associated experience of physicians in those settings. Some antibiotics are only available in secondary or tertiary hospitals due to high cost (e.g. vancomycin) or because they are restricted to use in centres that meet certain requirements as part of ASPs (e.g. piperacillin–tazobactam requires pre‐authorization of ID specialists in Serbia).

Finally, experts from 8 out of 14 countries (South Africa, Mexico, Brazil, Egypt, Kenya, Pakistan, Guatemala and Colombia) highlighted that supply chain issues impacted access to certain antibiotics (e.g. ceftazidime–avibactam, IV fosfomycin and cefepime). These issues were particularly prominent during the COVID‐19 pandemic. Concern over the quality of antibiotics (e.g. sub‐standard or falsified medicines) also limits availability in some countries (e.g. Georgia, Nigeria and Colombia). Price is an important factor for access, particularly in the public sector.

## 5 Discussion

### 5.1 Introductory Pathways for Novel Reserve Antibiotics

**FIGURE 1:**
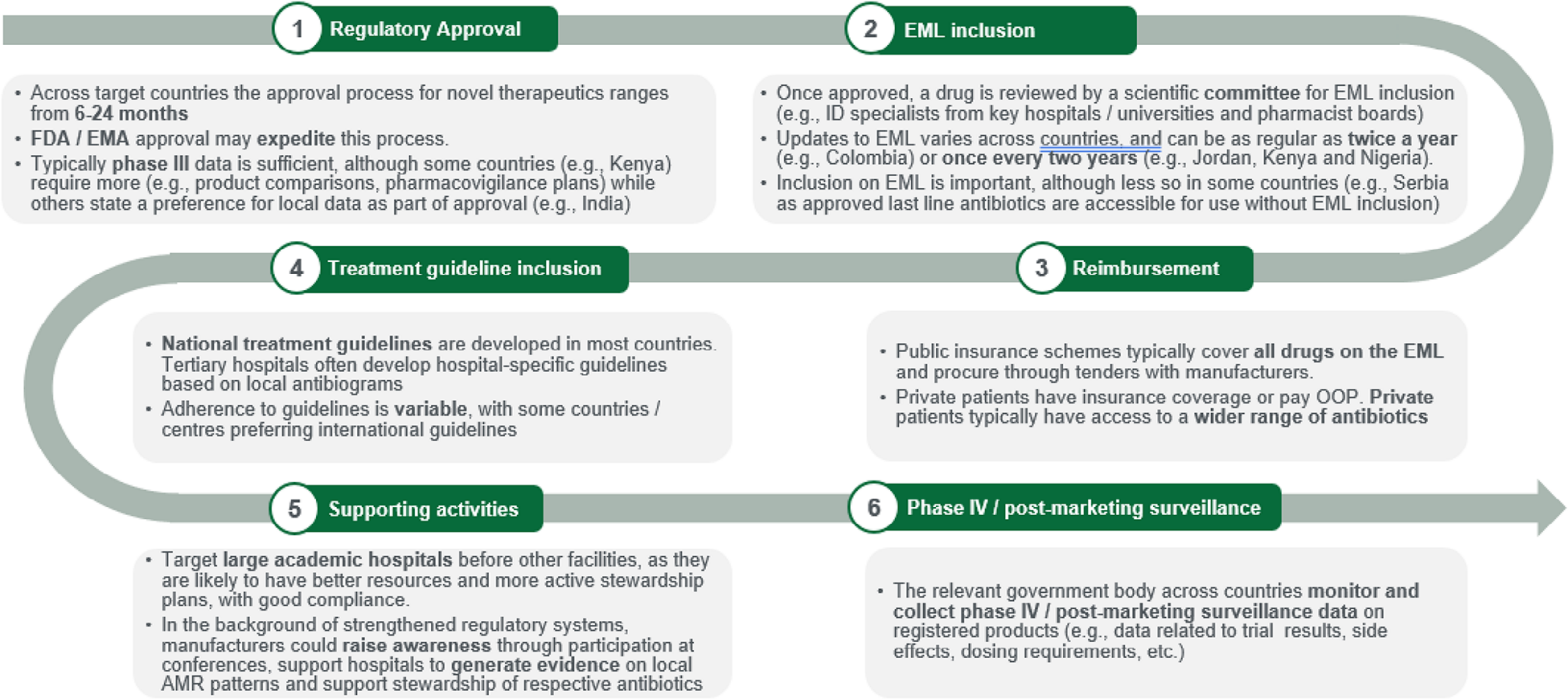
Introductory pathway for novel Reserve antibiotics. AMR, antimicrobial resistance; EML, Essential Medicines List.

Based on commonalities across 14 LMICs, review of national policies or guidance and interviewee input, new Reserve antibiotics in general may follow a six‐step introductory pathway (Figure 1). Although several steps in this pathway are common across medications, there are some that are specific to Reserve antibiotics.

#### 5.1.1 Regulatory Approval

The introductory pathway for novel Reserve antibiotics starts with regulatory approval at a national level (Figure 1). This process typically takes 6–24 months depending on the country. Across all target countries, prior US FDA and EMA approval accelerates timelines. Some countries also have additional data requests as part of the regulatory approval process. For example, the Central Drugs Standard Control Organization (CDSCO) in India accepts Phases I and II trial data generated in high‐income countries, but the Phase III data needs to be conducted in India or include Indians (if multinational) for approval, unless a specific waiver is granted.

### 5.2 EML Inclusion

A multidisciplinary panel (e.g. ID specialists from key hospitals/universities, pharmacist boards) then examines newly approved drugs across metrics such as the relevance of the disease area and degree of unmet need, formulation, quality, efficacy, safety and cost effectiveness for inclusion on the EML (Figure 1). EMLs can be updated as regularly as twice a year (e.g. Colombia) or once every 2 years (e.g. Jordan, Kenya and Nigeria). The importance of EML inclusion and the downstream impact this has on access to essential antibiotics is variable by country.

For example, in some countries, a newly approved drug is automatically included in the EML (e.g. Georgia). Others, for example, have multiple EMLs, one at the national level and others specifically for tertiary or quaternary centres (e.g. South Africa), public hospitals (e.g. the Cuadro Básico in Mexico) or even hospital‐specific EMLs (e.g. Panama). In other countries, physicians are not limited to prescribing drugs listed on the national EML (e.g. Serbia and India).

#### 5.2.1 Reimbursement

Across countries in this study, there are a range of reimbursement models (Figure 1). For example, in Serbia, around 80% of patients are publicly covered and drugs are directly reimbursed by the Republic Health Insurance Fund (RFZO), whereas most South Africans using the public sector are covered by the National Health Insurance scheme, which uses public funds to provide access to drugs on the EML to all citizens.

Public insurance schemes typically cover all drugs on the EML and procure through tenders with manufacturers. Patients accessing treatment through private insurance or co‐payments typically have access to a wider range of antibiotics, as they are not limited to those included on the EML.

#### 5.2.2 Treatment Guidelines Inclusion

The inclusion of novel Reserve antibiotics in treatment guidelines is an important step in the introductory pathway to ensure appropriate incorporation into clinical practice and longer term use (Figure 1). Among the 14 countries included in this study, 10 published national treatment guidelines related to antibiotic usage. In some cases, the national guidelines refer to the US or European guidelines, which, although useful, may not be optimized for local epidemiology. National guideline updates occur every 2 years on average and involve collaboration with physicians from leading tertiary hospitals, alongside other experts (e.g. epidemiologists). Additionally, in some countries like South Africa and Colombia, tertiary hospitals often develop hospital‐specific guidelines based on local cumulative antibiograms. In the absence of or, in some instances, instead of national guidelines, physicians refer to international guidelines (e.g. EU, WHO, CDC, IDSA, PAHO and ReLAVRA) to support their clinical decision‐making.

#### 5.2.3 Supporting Activities

Once a novel Reserve antibiotic gains approval, EML inclusion and features in national treatment guidelines, stakeholders, including MOH, experts and suppliers, can assist educational and data generation activities to support appropriate use and uptake in clinical practice (Figure 1). Targeting large academic hospitals before other facilities is beneficial, as they are likely to have better resources, improved ability to conduct AST in microbiology labs, and more active stewardship plans, with good compliance. The provision of prescribers’ training on how best to use novel Reserve antibiotics is also important to support long‐term availability and use. Disseminating key information, either directly through medical science liaisons or through conferences or educational events, to raise awareness of novel Reserve antibiotics and their appropriate use is important. Stakeholders can also support hospitals to generate evidence around local AMR patterns (including regularly updated cumulative antibiograms) and antibiotic use, highlighting the need for improved access to novel Reserve antibiotics. Lastly, introduction of apps to LMICs, similar to the Sanford Guide [18], with minimal or no subscription fees for better outreach and accessibility may support appropriate use.

#### 5.2.4 Phase IV/Post‐Marketing Surveillance

The relevant government body across countries (e.g. the National Agency for Food & Drug Administration Control in Nigeria) monitors and collects Phase IV/post‐marketing surveillance data on registered products (e.g. data related to trial results, side effects and dosing requirements) (Figure 1). Although this is a helpful tool to facilitate launch and appropriate use of novel Reserve antibiotics, their effectiveness can be variable (e.g. if only top centres are included in the study, this may not be a representative sample and could provide an inaccurate picture of real‐world use).

This article characterizes the AMR landscape and an introductory pathway for novel Reserve antibiotics across 14 LMICs, including clinicians’ and PHEs’ perspectives of the availability and barriers to access for key antibiotics. There are common barriers to accessing and appropriately using Reserve antibiotics faced across countries, including lack of availability of newer antibiotics, particularly those with activity against CR infections, some newer medicines being priced out of reach and lack of diagnostics to support both surveillance and cumulative local antibiograms and clinical management. In addition, antibiotic supply chains were often noted to be weak, potentially due to several reasons, including lack of information to inform forecasting or long procurement cycles. Across all countries included in this study, there is a need noted for government involvement to fund, lead and implement initiatives to tackle AMR. Access barriers may also be addressed by non‐governmental actors.

Affordability was noted to be a barrier to equitable access for many Reserve antibiotics. To improve affordability, originators may engage in public‐health oriented licensing with appropriate controls to support stewardship and to enable a more affordable supply and registration in LMICs [19]. Low and fragmented demand may also hamper price negotiations. More focused antibiotic formularies in public hospitals can help consolidate markets, leading to better buying power and easier forecasting, so long as there are antibiotics of sufficient spectrum to cover the resistance patterns encountered and some flexibility to access particular antibiotics in special cases through other mechanisms [20]. In addition, pooled procurement, at the national, regional or international level, may be employed to help improve negotiating power.

There are opportunities to strengthen the identified introduction pathway for antibiotics. Countries may identify antibiotics that address priority public health needs or priority pathogens and support more rapid registration or facilitate special import ahead of national regulatory approval to expedite access [21]. Recognizing the need for additional data to optimize antibiotic use, countries may develop a network of sentinel sites to help improve quality data collection in parallel with antibiotic introduction. Countries may also choose to help expand equitable access to Reserve antibiotics, especially in the public sector, by supporting public reimbursement of these medicines through including them on the EML or developing a limited‐use EML for reimbursement only for higher level health facilities, for example the South Africa National EML for Tertiary and Quaternary Level Health Facilities [22].

The introduction pathway may depend on baseline country criteria. Across the countries included in this study, variability was observed in the degree of national‐level support and guidance (e.g. guidelines and national ASPs), access to diagnostic and sensitivity testing, antibiotic access and availability, as well as other factors like level of awareness of AMR. Different archetypes were identified, ranging from countries with advanced AMR landscapes to those facing significant infrastructural or regulatory challenges; countries at different levels of preparedness can support different introductory pathways and invest in different ways to improve access. For example, in countries with national guidelines, national ASPs and access to advanced diagnostic and sensitivity testing, efforts could focus on initial introduction of Reserve antibiotics in top tertiary centres to build confidence and knowledge of appropriate use and then leverage local key opinion leader networks to support wider roll‐out.

This mixed‐method study was completed in 14 LMICs in Africa, the Americas, Asia and Europe through a combination of in‐depth qualitative interviews with 54 physicians and 17 PHEs, and a quantitative survey of 209 physicians. The study has important limitations which may limit its generalizability. Although efforts were made to avoid introducing bias into the sample, experts were recruited on the basis of their affiliation, evidence of participation in initiatives related to AMR (e.g. sponsors or stewards of NAPs or national ASPs) or participation in conferences or events related to AMR, authorship of AMR‐related peer‐reviewed publications or guidelines. As such, those included in this study were leading experts in their field and may have access to more resources than other clinicians and PHEs. Thus, their views may be biased and suggest better access to antibiotics, AMR diagnostics and antibiotic susceptibility testing in their countries versus their colleagues. For example, in Mexico, most public hospital laboratories have difficulties accessing basic consumables for antibiograms, and, although those that have in‐house research departments do have some state‐of‐the‐art equipment, it is rarely used for diagnostic purposes; as a result, most studies describing resistance prevalence in Mexico are based on more basic microbiological methods such as disk diffusion for AST [23, 24, 25].

The selection of the countries may also limit its generalizability. Although the countries represent a wide geographic scope, the included countries are middle‐income countries as middle‐income countries have higher burden of resistance patterns that may benefit from access to newer Reserve antibiotics. Low‐income countries likely have different health system contexts, and the findings from this study may not be as applicable.

Finally, the study did not specify whether the diagnostics and antibiotic susceptibility testing were available on site or by sample or patient referral. Thus, the findings may over‐represent access to diagnostics and antibiotic susceptibility testing capabilities, as there may be significant barriers to access off‐site testing capabilities.

## 6 Conclusion

Reserve antibiotics can be introduced appropriately across LMICs using a phased and monitored approach, working closely with local experts and documenting best practices.

Future research should be conducted to document pathways to optimize the introduction of needed antibiotics and demonstrate best practices to ensure their appropriate use, especially in LMICs, which are disproportionately impacted by AMR. Securing appropriate access to the required antibiotics should be the target for all countries to ensure equal access to life‐saving antibiotics.

## Author Contributions

J.C. and L.J. conceptualized the study. J.C., L.J., and T.L. contributed to the study instrument design, data acquisition and analysis. T.L. and J.C. drafted the manuscript. All authors contributed to interpretation of data, editing and review of the manuscript.

## Conflicts of Interest

F.M. has received honoraria for Pfizer for speaking engagement. M.V.V. has received educational grants and honoraria for speaking engagements from Pfizer, M.S.D., Biomerieux and West. All other authors have no conflicts of interest to declare.

## Data Availability

The data that support the findings of this study are available from the corresponding author, J.C., upon reasonable request.
